# Off-Pump Resection of Giant Intramural Left Ventricular Hydatid Cyst by Pleuropericardial Approach: a Case Report

**DOI:** 10.21470/1678-9741-2021-0197

**Published:** 2023

**Authors:** Dhiren Shah, Kishore Gupta, Dhaval Naik, Hiren Dholakia, Surabhi Madan

**Affiliations:** 1 Department of Cardiac Surgery, Care Institute of Medical Sciences (CIMS Hospital), Ahmedabad, Gujarat, India.

**Keywords:** Pleura, Cardiopulmonary Bypass, Anaphylaxis, Echinococcosis, Echinococcus, Pericardium, Heart Ventricles, Cysts

## Abstract

Primary cardiac hydatid cyst is a rare and fatal pathology, especially when involving the left ventricular free wall. A 44-year-old male was diagnosed with large intramural left ventricular hydatid cyst with wall thickness of 6 mm at the thinnest point. Cyst was accessed through pleuropericardial approach (left pleura opened, followed by entry into cyst directly through adjacent pericardium without removing the pericardial adhesions) which resulted in easy entry into the cyst, mitigating the risk of mechanical injury. This case report highlights that with detailed evaluation, cardiac hydatidosis can be addressed with off-pump technique, reducing the anaphylaxis risks and cardiopulmonary bypass-related effects.

## INTRODUCTION

Hydatid cyst disease or *echinococcosis* is a zoonotic disease caused by infection with the metacestode stage of the tapeworm Echinococcus. Clinical manifestation of echinococcosis depends on the involved organ along with number and size of the cysts^[1]^.

Cardiac component as a part of multivisceral involvement is observed in < 2% of cases, with primary infection of the heart being an exceedingly rare condition (< 0.2%). Isolated primary cardiac hydatidosis being rare can be mistaken for ventricular aneurysm, atrial myxoma, or simple epicardial cyst. Cardiac hydatid cysts can be pericardial, endocardial, or, very rarely, intramural. Left sided cysts usually tend to grow subepicardially whereas right sided cysts have tendency to grow subendocardially and intracavitarily^[2]^.

The symptoms range from being asymptomatic to having a life-threatening course. As in our case, cardiac hydatid disease presented with chest pain and shortness of breath. Additionally, palpitations and recurrent syncope may also occur and are related to underlying cardiac arrhythmias or mechanical effect. Intracardiac rupture of cyst can result in pulmonary embolism or stroke^[3,4]^. Release of cyst contents can induce a life-threatening allergic reaction, which might also be encountered during surgical excision of hydatid cysts^[5]^.

Surgical excision, usually with cardiopulmonary bypass (CPB) support, remains the mainstay of treatment even in asymptomatic patients due to risk of rupture, but excision needs to be therapeutically supplemented by anthelminthic medications in the preoperative and postoperative periods to prevent recurrences^[6,7]^.

The aim of our case report is to present a successful resection of giant left ventricular intramural hydatid cyst on beating heart and to outline important considerations during off-pump surgical excision.

## CASE REPORT

A 44-year-old non-diabetic, normotensive male presented with history of left sided chest pain and cough with progressive dyspnea for the previous two months. The patient was examined and initially sent for high-resolution computed tomography (HRCT) scan and echocardiography. HRCT revealed large well encapsulated multiloculated mass measuring around 13.9 × 10 × 12.9 cm in size within the myocardium of the left ventricular free wall along with mild calcification of medial wall. HRCT also showed the mechanical effect causing reduction in left ventricular intraluminal volume and diaphragmatic depression.

**Fig. 1:**
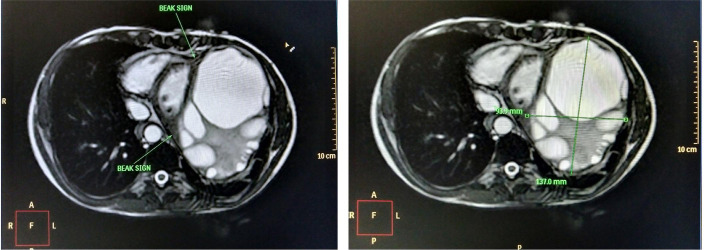
Transverse section on cardiac magnetic resonance imaging showing beak sign appearance indicating myocardial origin.

Cardiac magnetic resonance imaging (MRI) was done in order to delineate the extent and tissue penetration. MRI showed “beak sign” on the medial side of cyst indicating its myocardial origin and attenuated left ventricular wall thickness of 6-7 mm at the thinnest portion and 11-12 mm at the thickest portion. Reduction in left ventricular volumes was also noted (Figure 1). Coronary angiography was done to look for distortion of coronary anatomy and feeder vessel (if any) to the cyst, but it revealed normal epicardial coronaries. Immunoglobulin G echinococcal antibodies were found to be 16.8 units.

**Fig. 2:**
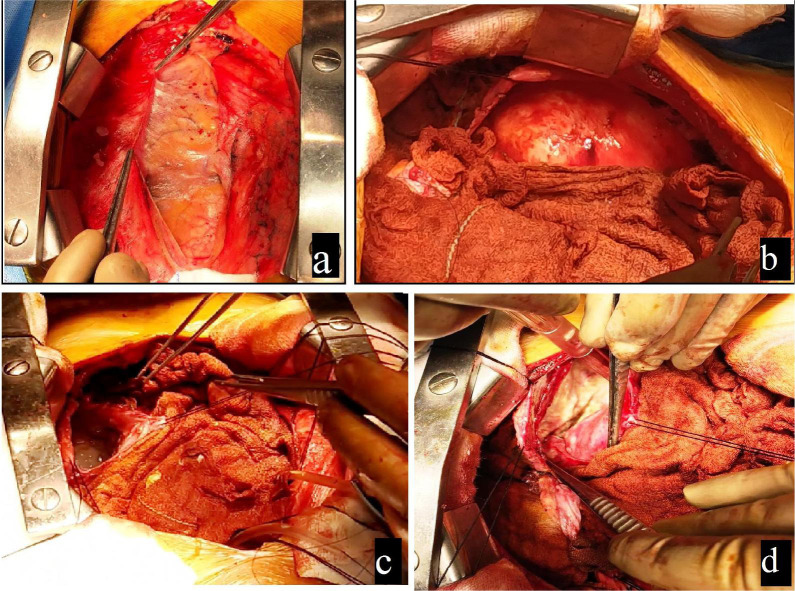
a) Intraoperative image showing adhesions over the epicardial surface; b) hydatid cyst seen bulging anteriorly and adhered to pericardium laterally; c) pleuropericardial approach showing entry into the cyst (stay sutures seen over cystic wall); d) large cystic cavity seen after emptying of contents.

**Fig. 3:**
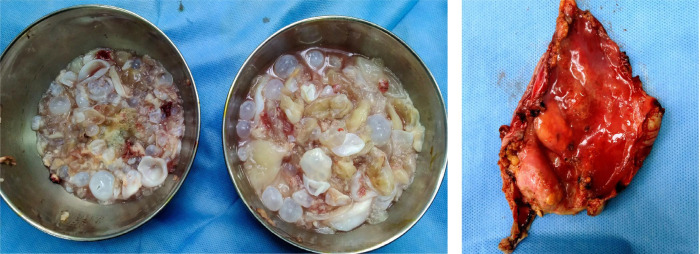
Daughter cysts, cystic contents, and resected cystic wall.

After two weeks of prior albendazole therapy, the patient was prepped for surgery. Sternotomy was done, and, as anticipated, the pericardium was found adherent to whole epicardial surface (Figures 2A and 2B). Adhesions were dissected superiorly and inferiorly. Due to dense adhesions over the lateral epicardial and cystic surface, left pleura was opened, and the cyst was approached from the lateral side (Figure 2C). Whole left lung and epicardial surfaces were covered with 10% betadine-soaked sponges. Purse string sutures were placed over the aorta. The cyst was punctured with 18 gauze needle syringe, and around 100 ml of clear fluid was aspirated to partially decompress the cyst initially. After confirmation, the cyst was incised over the same portion, and fluid was sucked out directly without spillage into the nearby surgical field. Stay sutures were placed over the cyst wall followed by removal of daughter cysts, cystic fluid, and inner layer of cyst (Figure 2D). Cavity was kept filled with betadine solution for two minutes. After removal of betadine, the lateral cystic wall was excised. Sponges were removed, and the whole pleural and pericardial cavity was washed with 1% betadine solution. Cyst wall and contents were sent for histopathology which showed eosinophilic lamellated cyst wall with presence of scolices and hooklets (Figure 3). The cyst wall showed marked fibrosis and dense chronic inflammation and congestion.

**Fig. 4:**
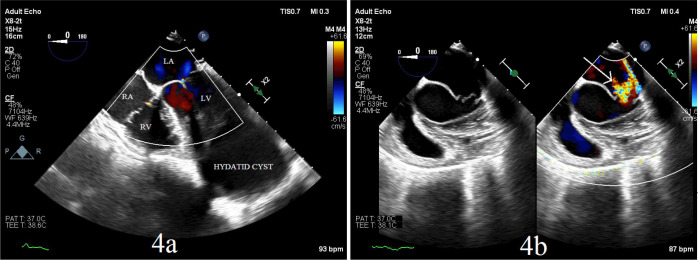
a) Transesophageal echocardiography image showing large left ventricular hydatid cyst; b) transesophageal echocardiography image after the resection of cyst showing mitral regurgitation (white arrow).

**Fig. 5:**
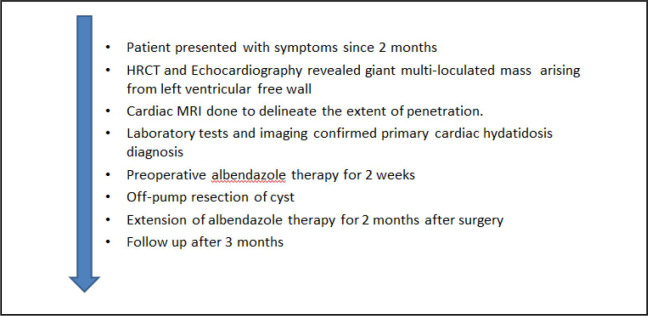
Historical and current information from the case report as episode of care organized in form of timeline. HRCT=high-resolution computed tomography; MRI=magnetic resonance imaging

After resection, echocardiography showed moderate mitral regurgitation possibly due to decompression of the ventricular cavity, but the patient remained hemodynamically stable (Figure 4). Postoperatively, the patient had mild mitral regurgitation. He was placed on albendazole for two months after discharge. After three months of follow-up, the patient is clinically stable and has mild mitral regurgitation on echocardiography (Figure 5).

## DISCUSSION

The heart is a rare but potentially fatal site for hydatid cyst, especially in the left ventricular free wall. A commonly involved site is the left ventricle, followed by right ventricle, interventricular septum, atria, and pulmonary artery. In advanced cases, rupture is a lethal complication of cardiac hydatid cyst, especially if not treated timely following the initial diagnosis or if the patient is presented belatedly.

We report our experience with a giant intramural left ventricular hydatid cyst which was successfully resected on beating heart. Cardiac intramural hydatid cyst is a rare entity, and its management should be based on multidisciplinary approach involving collaboration between surgeons, radiologists, and infectious disease specialists. To our knowledge, this is the largest and first intramural ventricular hydatid cyst removal done without CPB. Cardiac hydatid cyst resection is usually performed under CPB support, but detailed preoperative evaluation and careful intraoperative assessment can result in extirpation without CPB support. This can aid in avoiding the CPB-related effects and possible risk of anaphylactic shock had there been aspiration of fluid contents into the circulation.

Certain factors seemed pivotal for off-pump cyst resection with successful outcome:

- Left ventricular wall thickness ≥ 6 mm adjacent to cyst with no apparent breach in myocardial wall on preoperative diagnostic modalities and intraoperative transesophageal echocardiography assessment.
- Clear fluid on aspiration along with partial decompression of the cyst prior to incision helps in avoiding any inadmissible complication or spillage.
- Once the cyst has been cleared off its contents, use of protoscolicidal agent followed by excision of germinative layer and resection of free cyst wall.

Steady progression of the pathology and inflammation is the most likely reason for adhesions between cyst and pericardium. Pleuropericardial approach (entering into the cyst across the pericardium) mitigates the risk of mechanical injury and sudden rupture in an attempt to clear adhesions from pericardium. Covering the pleural and pericardial surface with betadine-soaked sponges before entering the cyst cavity helps in avoiding contamination.

## CONCLUSION

In conclusion, primary hydatid cyst of the heart, specifically the intramural type, is rare. Detailed and meticulous assessment should be considered in large ventricular hydatid cyst in order to delineate the extent and to choose the surgical strategy with lesser risks and invasiveness. We are reporting this case to underline that cardiac hydatidosis can be addressed with off-pump technique approach as compared to empty beating on-pump technique and thereby reducing the risk of anaphylaxis and CPB-related effects.
